# Therapeutic Tools for Vulvovaginal Candidiasis: Current and Emerging Antifungal Agents

**DOI:** 10.3390/jof12020152

**Published:** 2026-02-20

**Authors:** Guillermo Quindós, Iker De-la-Pinta, Cristina Marcos-Arias, Nerea Jauregizar, Elena Sevillano, Lucila Madariaga, Elena Eraso

**Affiliations:** 1 Department of Immunology, Microbiology and Parasitology, Faculty of Medicine and Nursing, University of the Basque Country (UPV/EHU), 48080 Bilbao, Spain; iker.delapinta@ehu.eus (I.D.-l.-P.); cristina.marcos@ehu.eus (C.M.-A.); elena.sevillano@ehu.eus (E.S.); lucila.madariaga@ehu.eus (L.M.); elena.eraso@ehu.eus (E.E.); 2 Instituto de Investigación Sanitaria Biobizkaia, 48903 Barakaldo, Spain; nerea.jauregizar@ehu.eus; 3 Department of Pharmacology, Faculty of Medicine and Nursing, University of the Basque Country (UPV/EHU), 48080 Bilbao, Spain

**Keywords:** vulvovaginal candidiasis, antifungal treatment, azoles, clotrimazole, fluconazole, miconazole, nystatin, ibrexafungerp, oteseconazole

## Abstract

Vulvovaginal candidiasis (VVC) represents a widespread gynaecological challenge, affecting approximately 75% of women at some point during their reproductive years, with a significant subset progressing to recurrent forms (RVVC). Classical azoles and polyenes remain the cornerstone of therapy. However, their clinical utility is undermined by the rise of azole-resistant non-*Candida albicans* species, the capacity of *Candida* to form biofilms, and a complex variety of host-related factors that complicate disease expression and therapeutic response. This narrative review provides a critical up-to-date examination of the therapeutic landscape, integrating current diagnostic algorithms with pharmacological strategies for both acute, recalcitrant and recurrent VVCs. We assess the efficacy and safety of established antifungal agents alongside the breakthrough introduction of novel drug classes, with a particular interest in the oral triterpenoid ibrexafungerp and the tetrazole oteseconazole, which offer new mechanisms of action for cases that fail to respond to standard regimens. Furthermore, we address the management of a special clinical scenarios, including pregnancy and lactation, and explore promising emerging innovative approaches such as mucoadhesive formulations, immunomodulatory approaches, and alternative non-antifungal therapies. Ultimately, this review aims to support clinical decision-making by balancing the accessibility and user-friendliness of conventional treatments with the targeted precision offered by modern antifungal agents.

## 1. Introduction

Vulvovaginal candidiasis (VVC) represents a significant global burden among genital infectious diseases: It is estimated that approximately 75% of women will experience at least one acute episode during their lifetime, and about half of these women eventually developing a second episode. VVC occurs predominantly during the reproductive years and affect 20–30% of pregnant women. Its pathophysiology is multifactorial, arising from a complex interplay between fungal virulence attributes and host susceptibility. Elevated oestrogen levels, whether occurring physiologically during pregnancy or induced by the use of high-dose oral contraceptive, increase vaginal glycogen concentration, thereby promoting *Candida* adhesion, germination, and hyphal development. Likewise, metabolic disorders, such as poorly controlled diabetes mellitus or immunosuppressive conditions, compromise the local vaginal defence mechanisms, enabling fungal overgrowth and tissue invasion. Behavioural practices, including the use of tight synthetic clothing or intrauterine devices, may further create a local microenvironment that is conducive to yeast proliferation (Table 1) [1,2,3,4,5,6].

Clinically, VVC is classified based on severity and relevant host factors. While most cases present as acute VVC (AVVC), a subset may be considered complicated and therefore require more specific diagnostic and therapeutic approaches. This category includes VVCs occurring in pregnant women, patients with poorly controlled diabetes mellitus, those with local or systemic immunodeficiencies, and those with infections caused by species other than *Candida albicans* [4,7,8,9,10,11,12]. In addition, VVCs coexisting with other genital infections, such as bacterial vaginosis or trichomoniasis, are also classified as complicated [13,14].

A particularly problematic form of the disease is recurrent VVC (RVVC), which occurs in 5–10% of women with AVVC. It is defined as two documented episodes within six months or more than three episodes per year, with microscopic confirmation in at least two episodes and culture-proven *Candida* isolation in one of them [1,3,4,10,13,15]. RVVC is estimated to affect 138 million women annually [16]. The management of RVVC poses a major challenge due to the recalcitrant nature of the infection and the significant psychological impact it has on affected patients. These patients are at greater risk of experiencing physical and emotional exhaustion, as well as disruption to their personal and professional activities, and a substantial decline in their overall quality of life. RVVC is often frustrating for both patients and healthcare professionals and is commonly managed inappropriately. Many women report feelings of shame, stigma, and medical neglect, which often lead to self-medication with over-the-counter products (OTC) or home remedies [7,17,18,19,20].

Despite the availability of classical antifungal agents, therapeutic failures are becoming increasingly common, driven by the emergence of azole-resistant isolates and the ability of *Candida* to form biofilms. This narrative review provides an updated overview of the current therapeutic landscape for VVC. We examine the aetiology and diagnostic considerations essential for effective management, discuss the pharmacology and clinical use of established agents, and critically evaluate recent therapeutic innovations, including the novel triterpenoid ibrexafungerp and the tetrazole oteseconazole, to assess clinical decision-making in both AVVC and RVVC.

## 2. Aetiology of Vulvovaginal Candidiasis

The species most frequently isolated in women suffering from VVC is *C. albicans* (75–95% of cases), although prevalence varies widely due to physiological, geographical, cultural, and socioeconomic factors. Isolates of *C. albicans* are usually susceptible to commonly used antifungal agents [3,5,21,22,23,24]. However, precise estimates of the prevalence of non-*C. albicans* species remain limited, as diagnostic testing is performed in fewer than 25% of women with suspected vaginal infection. Moreover, many clinicians only receive culture reports confirming the presence or absence of yeasts without species-level identification [3,4,15,22,25].

The most frequently isolated non-*C. albicans* species include *Candida glabrata* (also named *Nakaseomyces glabratus*, 3–15% of cases), *Candida parapsilosis* (4–10%), *Candida tropicalis* (1–2%), *Candida krusei* (also named *Pichia kudriavzevii*, 1%), *Candida guilliermondii* (also named *Meyerozyma guilliermondii*), *Candida africana*, *Candida bracarensis* (also named *Nakaseomyces bracarensis*), *Candida nivariensis* (also named *Nakaseomyces nivariensis*), *Candida metapsilosis*, *Candida orthopsilosis*, *Candida auris* (also named *Candidozyma auris*).

Although taxonomically distinct from the genus *Candida*, *Saccharomyces cerevisiae* is also occasionally identified as a cause of yeast vaginitis that is clinically indistinguishable from VVC [3,5,8,12,22,23,24,25,26,27,28,29,30,31,32,33]. The association between these species of yeasts and clinical symptoms remains unclear, although non-*C. albicans* isolates are more frequently isolated in older women and in those previously treated with fluconazole [11]. Some species show reduced susceptibility to fluconazole and itraconazole: approximately half of *C. glabrata* isolates are poorly susceptible or resistant to fluconazole, and *C. krusei* is intrinsically resistant [5,11,12,22,25,28,30]. Vaginal isolates of *C. guilliermondii*, *C. auris*, and *S. cerevisiae* are frequently multidrug-resistant [11,22,32,34,35,36,37].

## 3. Diagnosis of Vulvovaginal Candidiasis

Accurate diagnosis is essential to distinguish VVC from other vulvovaginal dermatoses and infectious vaginitis, thereby avoiding unwarranted empirical therapy. Although clinical presentation typically includes pruritus, burning sensation, erythema, and increased whitish vaginal discharge, these symptoms are non-specific and often overlap with bacterial vaginosis, trichomoniasis, or allergic reactions [1,3,4,38,39].

**Figure 1:**
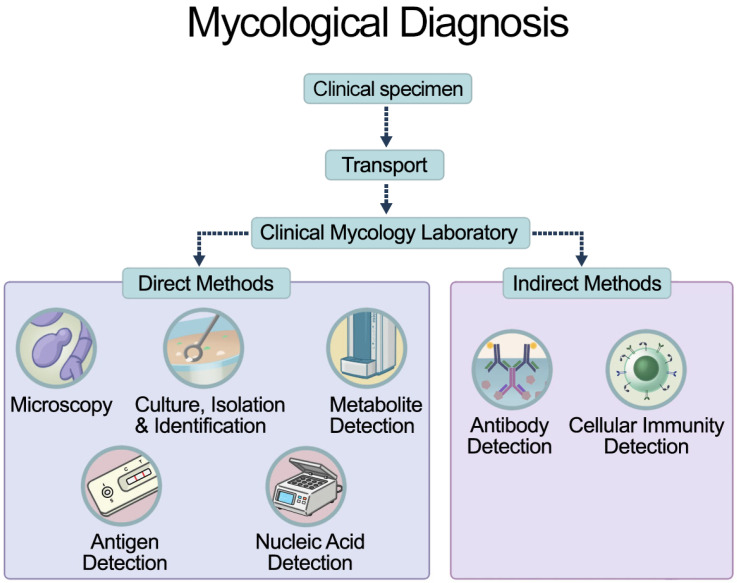
Mycological diagnosis of vulvovaginal candidiasis.

Consequently, diagnosis should not rely solely on clinical features but must be confirmed using wet mount microscopy (10% KOH), Gram staining, or culture. While microscopy provides an immediate result, its sensitivity is limited; therefore, mycological culture remains the gold standard, particularly for detecting non-*C. albicans* species in recurrent or complicated VVCs (Figure 1) [1,4,40,41,42,43,44,45].

Advances in diagnostics have introduced molecular techniques (PCR) and proteomic identification tools, such as MALDI-TOF (Matrix-Assisted Laser Desorption/Ionisation-Time-Of-Flight), which offer enhanced sensitivity and rapid species-level identification without the delay associated with traditional culture. Some molecular diagnostics are commercially available, such as Aptima *Candida*/*Trichomonas* Assay (Aptima, Hologic, San Diego, CA, USA), BD MAX Vaginal Panel and BD Affirm VPIII (Becton Dickinson, Franklin Lakes, NJ, USA), Seegene Allplex Vaginitis (Seegene, Seoul, Republic of Korea), or Vaginal Panel Real-Time PCR kit (Vircell, Santa Fe, Spain) [40,43,46,47]. However, their cost may limit routine implementation. It is important to note that the isolation of *Candida* should be interpreted in conjunction with clinical symptoms, as 20–30% of healthy women are asymptomatic carriers who do not require antifungal therapy [8,9,48,49].

Antifungal resistance should be verified by susceptibility testing before escalating therapy, since poor response is frequently attributable to an alternative or concurrent disease rather than true resistance. Where in vitro susceptibility testing is unavailable, species identification may help to predict resistance patterns. It is essential to recognise that no current treatment achieves complete and permanent eradication of *Candida*, and positive culture or PCR findings may simply reflect colonisation. Pre- and post-treatment cultures are therefore useful to rule out other causes of vulvovaginitis or for assessing resistance when clinical response is suboptimal. If symptoms persist despite evidence-based therapy and a subsequent negative culture, an alternative aetiology should be explored. VVC may be mistaken for bacterial vaginosis (caused by *Gardnerella vaginalis* and associated bacteria) or trichomoniasis; hence, microbiological documentation of each suspected VVC episode is essential to exclude other causes [13,14,21,39].

## 4. Therapeutic Strategies and Clinical Management of Vulvovaginal Candidiasis

**Figure 2:**
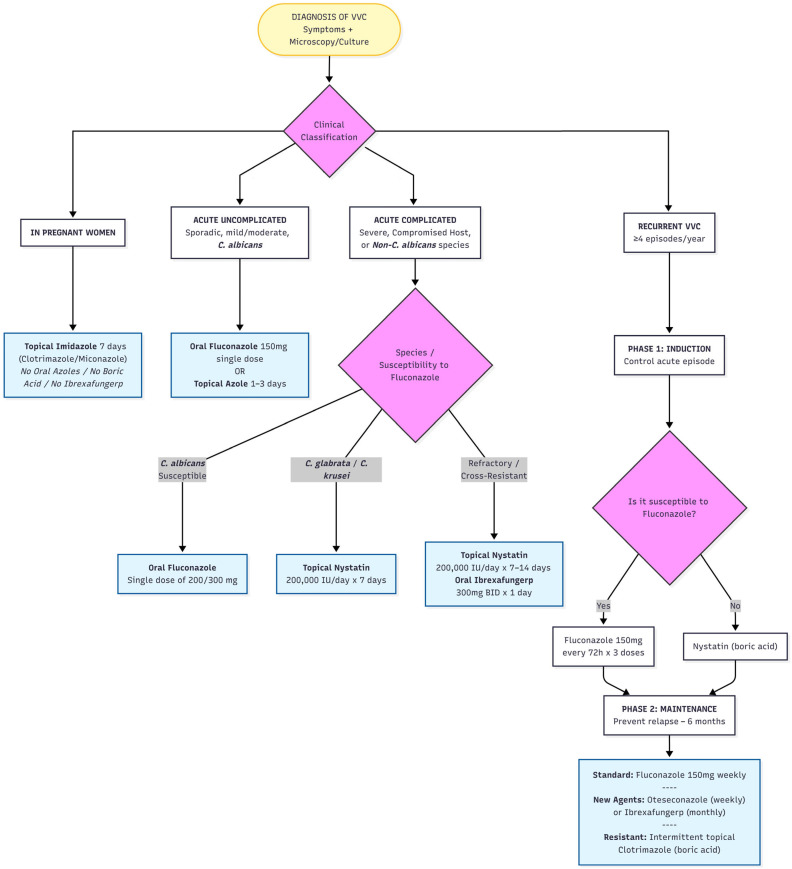
Therapeutic algorithm for VVCs according to their medical classification.

Current management of VVC relies on accurate classification of cases as either uncomplicated or complicated, as this distinction guides the selection of topical versus systemic therapy. Antifungal agents are categorised into four major classes: polyenes, azoles, echinocandins, and triterpenoids [50,51,52,53]. Although echinocandins (e.g., anidulafungin, caspofungin, micafungin and rezafungin) exhibit potent activity against *Candida* species, their use in the treatment of VVC is restricted by the requirement for intravenous administration. Consequently, azoles and polyenes remain the principal therapeutic options. Other drugs such as 5-fluorocytosine, terbinafine, or ciclopirox olamine are rarely used [36,54]. Echinocandins and broad-spectrum triazoles have a very limited role in refractory VVCs. Figure 2 shows a proposed therapeutic algorithm for vulvovaginal candidiasis [53,55].

### 4.1. Classical Antifungal Agents: Polyenes and Azoles

**Figure 3:**
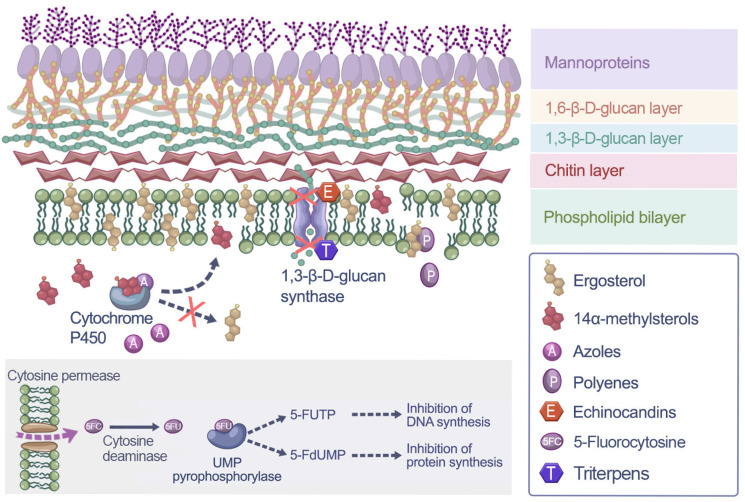
Schematic representation of the *Candida* cell wall architecture and targets of antifungal agents. The schematic illustrates the inhibition sites for the drug classes listed in the legend within the fungal cell wall and membrane. Regarding the intracellular pathway of 5-fluorocytosine, the prodrug is converted into 5-fluorouracil (5-FU). This metabolite is subsequently processed into 5-fluorouridine triphosphate (5-FUTP) and 5-fluoro-2′-deoxyuridine monophosphate (5-FdUMP), leading to the inhibition of protein and DNA synthesis, respectively.

Antifungal agents are pharmacologically classified into distinct families; however, in clinical practice their selection is guided by the route of administration and the severity of the infection. Polyenes (nystatin and amphotericin B) exert their effect by binding to ergosterol within the fungal cell membrane, forming pores that disrupt membrane integrity and ultimately lead to cell death (Figure 3).

Nystatin is not absorbed from the gastrointestinal tract or the vaginal mucosa, making it a safe option for localised treatment, particularly during pregnancy and lactation. Nonetheless, it is less convenient than azoles, often requiring 14 days of treatment to achieve comparable cure rates [50,51,52,53]. Amphotericin B is rarely used to treat uncomplicated VVC, although it may be formulated into vaginal suppositories for infections caused by non-*C. albicans* species.

Azoles inhibit the fungal cytochrome P450-dependent enzyme lanosterol 14-α-demethylase, thereby blocking ergosterol biosynthesis. This class is subdivided into imidazoles (mostly topical, such as clotrimazole, miconazole, econazole, butoconazole, fenticonazole, isoconazole, omoconazole, oxiconazole, sertaconazole, tioconazole), triazoles (mostly systemic, such as fluconazole, isavuconazole, itraconazole, posaconazole, terconazole, voriconazole) and tetrazoles (mostly systemic, such as oteseconazole) [56,57].Topical imidazoles, such as clotrimazole, miconazole, econazole, sertaconazole, etc., are the most widely used drugs for uncomplicated AVVC. They are available in various formulations, including creams, vaginal tablets, and ovules.

Clotrimazole, miconazole, and tioconazole show good in vitro activity against *Candida*, though less effective against *C. glabrata* and *C. krusei*. Treatment regimens include single high doses or lower concentrations for three consecutive nights [10,12,50,51,53,58,59]. Other topical azoles include bifonazole, eberconazole, fenticonazole, flutrimazole, oxiconazole, sulconazole, and terconazole. Topical application may cause mild irritation (<5%) [15,51,52,57,58,59,60,61,62]. Sertaconazole, a benzothiophene imidazole, is particularly noteworthy for its pronounced lipophilicity, which facilitates prolonged retention in the vaginal mucosa, and its intrinsic anti-inflammatory activity, which may contribute to a more rapid symptomatic relief [60]. Recent pharmaceutical advances include the development of sertaconazole-loaded mucoadhesive liposomes, which have demonstrated substantially improved vaginal retention and enhanced antifungal efficacy compared with conventional formulations [63].

Oral fluconazole is the only systemic azole licensed for routine VVC treatment. It is highly effective against *C. albicans*, with a single 150 mg dose achieving therapeutic vaginal concentrations lasting for at least 72 h (see Table 2 for detailed dosing regimens) [19,23,62,64]. Comparative studies indicate no significant difference in either clinical or mycological cure rates between oral fluconazole and topical imidazoles with both achieving cure rates exceeding 80–90% in uncomplicated cases. Consequently, the choice of treatment often depends on patient preference, cost consideration, and safety profile. Topical agents may cause mild local burning in fewer than 5% of cases, whereas oral fluconazole is associated with a low risk of gastrointestinal intolerance and drug–drug interactions. Fluconazole is generally avoided during pregnancy due to concerns regarding teratogenicity, particularly at higher doses [1,4,10,12,39,53,65].

### 4.2. Management of Complicated and Recurrent VVC (RVVC)

Complicated VVC, including infections caused by non-*C. albicans* species or occurring in immunocompromised hosts, requires prolonged therapy. In RVVC, the standard therapeutic approach comprises an induction phase to achieve clinical remission, followed by a maintenance regimen aimed at preventing relapse (Table 2).

Oral fluconazole (150 mg every 72 h for three doses) is the preferred induction regimen. For maintenance therapy, weekly fluconazole (150 mg) for six months significantly reduces recurrence rates, with over 90% of patients remaining disease-free while on therapy [12,60,64. Nonetheless, recurrences are frequent after therapy has been topped. Regarding therapeutic endpoints, although clinical cure, defined as resolution of symptoms, is sufficient for AVVC, mycological monitoring by culture is recommended in RVVC after completing the induction phase and periodically throughout maintenance in order to ensure sustained suppression of the fungal burden.

In cases of fluconazole resistance or clinical refractoriness, most commonly associated with *C. glabrata* or *C. krusei*, alternative agents become necessary. Oral itraconazole may serve as a second-line systemic agent, administered either as 200 mg twice in a single day or 200 mg once daily for three days, although its absorption is notoriously variable. Voriconazole and posaconazole, two oral and systemic broad-spectrum triazoles with reliable activity against fluconazole-resistant isolates, may be employed in more recalcitrant presentations, their use generally tempered by concerns regarding toxicity, drug–drug interactions and cost [24,31,35].

Isavuconazole is structurally related to fluconazole and voriconazole and provides one of the widest spectra among the triazoles [24,51,52,66]. It exhibits high oral absorption that is not influenced by food intake, gastric pH or mucositis. It has a large volume of distribution with high plasma protein binding. Compared with other azoles, it shows fewer drug interactions, which facilitates clinical handling and constitutes its principal advantage [53]. Another azole antifungal, albaconazole, is under investigation in single-dose oral regimens, aiming to improve treatment adherence and reduce recurrence rates, although it remains in the clinical evaluation phase [42,67]

For VVC caused by *C. glabrata* or other azole-resistant isolates, topical boric acid, delivered intravaginally at 600 mg once daily for fourteen to twenty-one days, remains a highly effective alternative; however, it is strictly contraindicated during pregnancy [4,11,22,39].

Special consideration must be given to pregnancy, as systemic azoles are contraindicated owing to their potential association with spontaneous abortion and congenital malformations. In pregnant women with VVC, the recommended standard of care consists of a topical imidazole, such as clotrimazole or miconazole, applied for 7 days. In cases that prove refractory during pregnancy, specialist consultation is advised in order to balance the benefits of alternative topical regimens, including extended courses of nystatin, against any potential risks to the mother and foetus.

### 4.3. New Antifungal Agents as Alternatives to Those in Common Use

The limitations of classical azoles have been the focus of recent therapeutic innovations particularly the challenges posed by resistance and drug–drug interactions. In this context, two novel agents, ibrexafungerp and oteseconazole, represent significant advances in the management of complex VVC, expanding the therapeutic landscape for VVCs that have traditionally been difficult to treat.

Ibrexafungerp belongs to a new antifungal class, the triterpenoids, derived from structural modifications of enfumafungin. It inhibits 1,3-β-D-glucan biosynthesis, exhibits a broad spectrum of activity against *Candida*, including *C. glabrata*, *C. krusei* and *C. auris*, and demonstrates efficacy against fungal biofilms [27,30,34,35,68]. Although structurally distinct from echinocandins, it shares a similar spectrum of activity, retaining efficacy against *Candida* isolates resistant to azoles and most isolates resistant to echinocandins. Partial overlap in binding sites with echinocandins likely contributes to the low risk of cross-resistance. Its activity is fungicidal in vitro and is enhanced by the acidic vaginal pH characteristic of VVC [34].

Ibrexafungerp has favourable oral bioavailability, with absorption enhanced by food and reduced by acid-secretion inhibitors and distributes widely into tissues. It is highly lipophilic, >99% protein-bound, and is primarily eliminated in faeces. Pharmacologically, it acts as a CYP3A4 substrate and a reversible CYP2C8 inhibitor, though the overall risk of clinically relevant drug interaction appears low. Adverse events are usually mild and gastrointestinal in nature (nausea, vomiting, abdominal pain, diarrhoea), and no clinically meaningful QT prolongation has been observed. Animal studies have indicated potential embryotoxicity; thus, its use is not recommended during pregnancy [51,69,70,71].

A regimen of 300 mg twice in a single day for the treatment of VVC is well tolerated and achieves clinical outcomes comparable to fluconazole. In the DOVE phase-2 clinical trial, the single-day 300 mg twice regimen was identified as optimal, with day-10 clinical cure rates comparable to fluconazole (51.9% vs. 58.3%). The benefits persisted to day 25, with fewer symptoms (70.4% vs. 50%) and reduced need for rescue medication (3.7% vs. 29.2%) [72,73]. Efficacy was further confirmed in the multicentre, randomised, placebo-controlled VANISH 303 and 306 phase-3 studies, in which ibrexafungerp was superior to placebo for clinical cure (50.5% vs. 28.6%; 63.3% vs. 44%), mycological eradication (49.5% vs. 19.4%; 58.5% vs. 29.8%), and sustained symptom resolution [74,75,76].

For recurrence prevention, the phase-3 double-blind CANDLE study enrolled women with RVVC. Following standard fluconazole therapy for the acute episode and the assessment of clinical response, participants were randomised to receive ibrexafungerp or placebo for prophylaxis. Ibrexafungerp 300 mg twice every four weeks for six cycles significantly reduced clinical recurrences (65.4% recurrence-free vs. 53.1% with placebo) and mycological recurrences (70.8% vs. 58.5%), with mostly mild adverse events (headache 20%, gastrointestinal symptoms 18.45%) [77,78]. The VANQUISH study is evaluating its role in complicated VVC, with preliminary findings indicating that monthly single-day dosing over six months significantly reduced RVVC recurrences [39].

Oteseconazole, a tetrazole characterised by a prolonged half-life of approximately 138 days, has been developed for the treatment and prevention of RVVC. It has enhanced selectivity for fungal CYP51 while reducing off-target activity against human enzymes, thereby improving potency and tolerability. It shows high activity against *Candida*, including fluconazole-resistant isolates [52,56,79,80,81,82].

In the ultra-VIOLET phase-3 trial, oral oteseconazole (600 mg on day 1 and 450 mg on day 2, followed by weekly maintenance starting on day 14) demonstrated efficacy comparable to fluconazole in resolving the initial episode (93.2% versus 95.8%) and superiority over placebo in preventing subsequent recurrences (5.1% versus 42.2%), with an adverse-event profile similar to that of fluconazole. In vitro, oteseconazole is approximately 40-fold more potent than fluconazole against most *Candida* species [12,51,79,80].

In RVVC, the licensed regimen consists of a two-day induction phase followed by eleven weekly maintenance doses. Across trials, up to 95% of patients remained recurrence-free in the long term, compared with approximately 60% in those treated with fluconazole. Notably, most participants had infections due to *C. albicans*, and evidence for non-*C. albicans* species remains limited. A major limitation is its contraindication in women of childbearing potential due to the risk of foetal toxicity and the prolonged exposure window—estimated at around 690 days—requiring strict contraceptive measures and careful patient selection in routine clinical practice [39,51,64,79]. However, despite their clinical advantages, the broader uptake of both ibrexafungerp and oteseconazole remains constrained by their substantially higher cost relative to generic azoles, such as fluconazole, as well as by their limited availability in resource-restricted settings.

### 4.4. Non-Antifungal Alternative Therapeutic Strategies

Given the enduring challenges posed by resistance and recurrence, the focus has increasingly shifted towards non-antifungal therapeutic strategies, including immunomodulatory approaches, antifungal peptides, selected probiotic strains, and other non-conventional interventions, such as traditional or plant-derived remedies [83,84,85,86,87,88,89]. Additional formulations with antifungal activity may be used in combination therapy for VVC. These include chlorhexidine, povidone-iodine, gentian violet solutions, potassium permanganate, methylene blue, sodium thiosulphate, propylene glycol, selenium sulphide, boric acid, and caffeic acid derivatives [84]. However, many chemical agents (such as acetic acid, povidone-iodine, boric acid) and natural products (propolis, garlic, lactoferrin, tea derivatives) lack robust clinical evidence and have not been evaluated in well-designed trials [4,12,84,90]. In this context, many patients turn to traditional remedies and natural products (e.g., garlic, tea tree oil, yogurt instillation) in an attempt to alleviate symptoms. Although certain essential oils have demonstrated in vitro antifungal activity, the clinical evidence supporting the efficacy and safety of such therapies remains limited when compared with conventional antifungal agents [91]. Clinicians should routinely inquire about the use of these products in order to avoid potential drug interactions or local mucosal irritation.

The use of monoclonal antibodies, cytokine-based therapies, or vaccines may provide valuable adjuncts to the management of VVC [38,92,93,94,95,96,97,98]. Two vaccines are in advanced stages of development, both undergoing phase-II clinical trials. The first, PEV7 (also known as Pevion7), is a virosomal vaccine incorporating Sap2 antigens and designed to prevent RVVC. It has demonstrated strong immunogenicity, favourable tolerability, and a significant reduction in recurrence rates, although regulatory approval has not yet been granted [39,99]. The second candidate, NDV-3A, is a recombinant vaccine based on the *C. albicans* Als3 protein fused to human albumin. Clinical trials have shown that it significantly decreases the number of VVC episodes and prolongs recurrence-free intervals. Preclinical studies have also demonstrated its capacity to inhibit biofilm formation and prevent systemic dissemination, providing further evidence of its potential as prophylactic and therapeutic strategy [100]. A more recent investigational vaccine, Candi5V, is currently enrolling participants for a phase-I/II human trial. This pentavalent bioconjugate vaccine aims to assess safety, immunogenicity, and preliminary efficacy in women with RVVC. These vaccine platforms are designed to elicit robust Th1- and Th17-mediated responses, thereby enhancing mucosal immunity and reducing the likelihood of recurrence. Although none has yet been approved for clinical use, they show considerable potential, particularly in patients with immunodeficiency or metabolic vulnerability, such as women with diabetes mellitus, by counteracting local immune dysfunction [39,96,97,101].

In gynaecology, probiotics are employed to maintain a healthy balance of the vaginal microbiota, a key component in the management of VVCs and bacterial vaginosis [86]. Current research focuses predominantly on *Lactobacillus* species, which help to protect the vaginal environment by producing lactic acid, thereby reducing the pH to between 3.5 and 4.5 and inhibiting the proliferation of pathogenic organisms [86,87,88]. These bacteria may also stimulate the production of bacteriocins and other antimicrobial peptides, which impede pathogenic colonisation through mechanisms of competition and exclusion. The use of *Lactobacillus vaginalis* is currently being investigated in clinical trials aimed at restoring vaginal microbiota homeostasis and preventing VVC in young women. Another therapeutical strategy combines *Lactobacillus* with boric acid [90]. Moreover, formulations based on prebiotics and postbiotics —non-viable microorganisms or their soluble metabolites— are under development to counteract vaginal dysbiosis associated with candidiasis [39,101].

**Table 2:** Antifungal treatment of vulvovaginal candidiasis [1,2,3,4,11,17,19,21,39,50,63,72,79,102].

Antifungal Agents	Vulvovaginal Candidiasis					OTC	Pregnancy **	SR and QE
	Acute			Recurrent				
	Uncomplicated	Complicated		Induction Therapy	Maintenance Therapy			
		Underlying Risk Factors	Fluconazole-Resistant *Candida* *					
	Presentation (Dose)							
Topical imidazole				Vaginal, for 14 days		Yes	Yes	1–2
Clotrimazole	Vaginal tablet, 500 mg in a single dose Vaginal tablet 200 mg/day or cream 2%, 5 g/day for 3 consecutive nights	Vaginal cream 1%, 5 g/day for 7–14 consecutive nights		Vaginal tablet, 100 mg/day for 14 days	Vaginal tablet, 500 mg weekly for 6 months	Yes	Yes	1–2
Miconazole	Vaginal ovule, 1200 mg in a single dose Vaginal ovule, 200 mg/day for 3 consecutive nights	Vaginal cream 2%, 5 g/day for 7 consecutive nights				Yes	Yes	1–2
Fluconazole	Oral capsule, 150–200 mg in a single dose	Oral capsule, 150 mg, every 72 h, 2–3 doses		Oral, 150 mg/day every 72 h (days 1, 4, and 7)	Oral, 150 mg or 200 mg weekly for 6 months	No	No	1
Tioconazole	Vaginal ointment 6.5%, 5 g in a single dose					Yes	Yes	1
Itraconazole	Oral capsules, 200 mg, twice in a single day Oral capsules, 200 mg/day, for 3 consecutive days				Oral tablet, 200 mg twice a week for 6 months	No	No	2–3
Butoconazole		Vaginal cream 2%, 5 g in a single dose	Vaginal cream 2%, once weekly for 2–3 weeks			No	Yes	2
Ibrexafungerp	Oral capsule, 300 mg, twice in a single day				Oral capsule, 300 mg, twice in a day each 4 weeks for 6 months	No	No	1–2
Terconazole	Vaginal cream 0.8%, 5 g/day for 3 consecutive nights Vaginal ovule, 80 mg/day for 3 consecutive nights	Vaginal cream 0.4%, 5 g/day for 7 consecutive nights	Vaginal cream 0.4%, 5 g/day for 14 consecutive nights			No	Yes	1
Oteseconazole ***				Oral, 600 mg on day 1, 450 mg on day 2	Oral, 150 mg weekly for 10 weeks, starting on day 14	No	No	1–2
Econazole	Vaginal pessary, 150 mg, twice in a single day Vaginal pessary, 150 mg/day for 3 consecutive nights					No	Yes (2nd/3rd trim) * Avoid in 1st trimester	1
Fenticonazole	Vaginal capsule, 600 mg in a single dose Vaginal capsule, 200 mg/day for 3 consecutive nights					No	Yes	1
Isoconazole	Vaginal pessary, 600 mg in a single dose Vaginal pessary, 150 mg/day for 3 consecutive nights					No	Yes	1
Sertaconazole	Vaginal ovule, 300 mg in a single dose or vaginal cream 2%, 5 g/day for 7 consecutive nights	Vaginal cream 2%, 5 g/day for 7 consecutive nights		Vaginal cream 2%, 5 g/day for 14 days	Vaginal ovule, 300 mg weekly for 6 months	No	No	1
Nystatin	Vaginal tablet, 200,000 IU/day for 7 consecutive nights Vaginal tablet, 100,000 IU/day for 14 consecutive nights				Vaginal tablet, 100,000 IU two or three times a week for six months	Yes	Yes	1
Amphotericin B	Vaginal suppositories, 50–100 mg/day for 14–21 consecutive nights Vaginal cream 3%, 5 g/day for 14 consecutive nights					Yes	Yes	3
Amphotericin B + 5-fluorcytosine			Vaginal cream, daily for 14–21 consecutive nights			No	No	3
Boric acid			Vaginal capsule, 600 mg/day, for 14–21 consecutive nights		Vaginal capsule, 600 mg/day for 14 days	Yes	No	2

* Antifungal in vitro testing recommended, ** Pregnancy and Breast feeding, *** Not for use in women with reproductive potential, IU = International Units, OTC = Over-The-Counter treatment, SR and QE = Strength of Recommendation and Quality of Evidence (1: Societies strongly support a recommendation for use based in properly designed clinical trials, 2: Societies and respected authorities support a recommendation for use, 3: Current and new drugs that need more studies for being recommended.

## 5. Future Directions and Therapeutic Priorities

Among the most pressing priorities in the field of fungal infections is the research of novel antifungal agents with the therapeutic potential, including antimicrobial peptides, new azoles, echinocandins, triterpenoids, natural products, and reformulations of existing antifungal compounds, alongside the development of both therapeutic and preventive vaccines [83,88,90,94,96,97]. It is likewise essential to re-examine current therapeutic approaches and assess whether recent advances in diagnostics my allow alternative management strategies [40,41,103].

Ibrexafungerp and oteseconazole represent promising alternatives to fluconazole for the treatment of RVVC, offering more convenient dosing schedules (monthly or weekly for three months). Both agents are currently under evaluation in clinical trials exploring wider clinical applications [65,71,78,81,104]. Oteseconazole has demonstrated its effectiveness in RVVC, achieving lower recurrence rates compared to previous treatment regimens [80,105]. However, neither agent is indicated during pregnancy, thereby limiting their use in this vulnerable patient population. In addition, the FDA has restricted oteseconazole to women who are not of childbearing age, owing to concerns regarding embryotoxicity and its prolonged half-life and exposure window [76,79,105]. Despite their clinical advantages, the broader implementation of these novel agents remains limited by their high cost and restricted availability across many healthcare settings, particularly in low-resource environments.

Future challenges for topical imidazoles include the development of improved galenic formulations capable of achieving higher in situ drug concentrations —for example, sprays, gels, liposomes, nanoparticles, and mucoadhesive systems— without increasing adverse effects. Notably, sertaconazole-loaded mucoadhesive liposomes have demonstrated enhanced vaginal retention and antifungal activity in preclinical models, representing a promising strategy for reducing treatment duration [63]. Additionally, the co-formulation of topical imidazole with anti-inflammatory and/or analgesic agents (such as diclofenac or lidocaine) is being explored to promote faster and more effective clinical resolution [39,59,85,106].

Given the challenges posed by resistance and recurrence, research into non-antifungal therapies is increasingly expanding and encompass immunotherapy, probiotics, and traditional remedies. Additional research strategies include the use of antifungal peptides and other non-conventional approaches [83,85,87,89,90,91,95,96,97,99,100,107]. In this context, many patients turn to traditional medicine and natural products, such as garlic, tea tree oil, yogurt instillation, in an attempt to relieve symptoms. Although certain essential oils have shown antifungal activity in vitro, robust clinical evidence supporting their efficacy and safety remains limited when compared to conventional antifungal treatments [91].

## Figures and Tables

**Table 1:** Host-related and behavioural risk factors that predispose to vulvovaginal candidiasis [1,2,3,4,5,6].

Host’s Predisposing Factors		Host’s Behaviours	
Risk	Mechanisms	Risk	Mechanisms
Genetic factors	Immune-related polymorphisms reduce antifungal defences	Tight clothing	Increases perineal moisture and temperature; may trigger hypersensitivity
Diabetes mellitus/corticosteroid therapy/Obesity	Hyperglycaemia and immunosuppression impair mucosal immunity Increased inflammation and altered glucose metabolism favour *Candida* proliferation	Poor hygiene	Accumulation of moisture, sweat and secretions creates a favourable environment for *Candida* growth
Pregnancy	Elevated oestrogen enhances adhesion, germination and hyphal formation; reduced local immunity	Intimate hygiene practices	Showering, perfumed soaps and wipes disrupt microbiota and irritate mucosa
Antibiotic treatment	Loss of lactobacilli leads to dysbiosis and increased vaginal pH	Sexual behaviour	Mechanical friction and transient pH shifts cause microdamage and facilitate *Candida* adherence Oral–genital contact and glycerin-based lubricants alter pH and introduce non-vaginal microbiota Use of spermicides and condoms alter vaginal microbiota; *Candida* can metabolise spermicidal compounds
Immunodeficiency	Impaired mucosal immune responses	Lifestyle habits	High-sugar diet, stress, sleep deprivation and prolonged moisture favour *Candida* proliferation

## Data Availability

No new data were created or analyzed in this study. Data sharing is not applicable to this article.

## Abbreviations

The following abbreviations are used in this manuscript:

AUC	Area Under the Curve
AVVC	Acute Vulvovaginal candidiasis
CLSI	Clinical and Laboratory Standards Institute
Cmax	Maximum Concentration
EUCAST	European Committee on Antimicrobial Susceptibility Testing
FDA	US Drugs and Foods Administration
MALDI-TOF	Matrix-Assisted Laser Desorption/Ionisation-Time-Of-Flight
MIC	Minimum Inhibitory Concentration
OTC	Over-The-Counter treatment
PCR	Polymerase Chain Reaction
PK-PD	Pharmacokinetics and Pharmacodynamics
PoC	Point of Care
QE	Quality of Evidence
RVVC	Recurrent Vulvovaginal Candidiasis
SR	Strength of Recommendation
VVC	Vulvovaginal Candidiasis
